# Precocious puberty in a girl with 3-methylglutaconic aciduria type 1 (3-MGA-I) due to a novel *AUH* gene mutation

**DOI:** 10.1016/j.ymgmr.2020.100691

**Published:** 2020-12-02

**Authors:** Neli Bizjak, Mojca Zerjav Tansek, Magdalena Avbelj Stefanija, Barbka Repic Lampret, Ajda Mezek, Ana Drole Torkar, Tadej Battelino, Urh Groselj

**Affiliations:** a Department of Child, Adolescent and Developmental Neurology, University Children's Hospital, University Medical Centre Ljubljana, Bohoriceva 20, Ljubljana, Slovenia; b Department of Endocrinology, Diabetes and Metabolic Diseases, University Children's Hospital, University Medical Centre Ljubljana, Bohoriceva 20, 1000 Ljubljana, Slovenia; c Faculty of Medicine, University of Ljubljana, Vrazov trg 2, 1000 Ljubljana, Slovenia; d Clinical Institute for Special Laboratory Diagnostics, University Children's Hospital, University Medical Centre Ljubljana, Bohoriceva 20, 1000 Ljubljana, Slovenia; e Unit for Clinical Dietetics, University Children's Hospital, University Medical Centre Ljubljana, Bohoriceva 20, 1000 Ljubljana, Slovenia

**Keywords:** 3-MGA-I, 3-methylglutaconic aciduria type 1, Precocious puberty, *AUH* gene, GnRH agonist, Triptorelin, 3-HIVA, 3-hydroxyisovaleric acid, 3-MGA-I, 3-methylglutaconic aciduria type I, 3-MG, 3-methylglutaric acid, 3-MGH, 3-methylglutaconyl-CoA hydratase, C5-OH, 3-hydroxyisovaleryl-carnitine, GnRH, Gonadotropin-releasing hormone, IEM, inborn errors of metabolism, LC-MS/MS, Tandem mass spectrometry, LH, luteinizing hormone, MRI, Magnetic resonance imaging, NBS, newborn screening, ToL, The Tower of London test, UCHL, University Children's Hospital Ljubljana

## Abstract

3-methylglutaconic aciduria type 1 (3-MGA-I) (MIM ID #250950) is an ultra-rare, autosomal recessive organic aciduria, resulting from mutated *AUH* gene, leading to the deficient 3-methylglutaconyl-CoA hydratase (3-MGH). Only around 40 cases are previously reported, caused by a spectrum of 10 mutations.

The clinical spectrum of 3-MGA-I in children is heterogeneous, varying from asymptomatic individuals to mild neurological impairment, speech delay, quadriplegia, dystonia, choreoathetoid movements, severe encephalopathy, psychomotor retardation, basal ganglia involvement. Early dietary treatment with leucine restriction and carnitine supplementation may be effective in improving neurological state in pediatric patients with 3-MGA-I.

We presented a girl with 3-MGA-I due to novel *AUH* gene mutation (homozygous variant c.330 + 5G > A) and confirmed by almost undetectable 3-MGH-enzyme activity, who initially presented with central precocious puberty at an early age of 4.5 years.

Precocious puberty might be associated with the 3-MGA-I, as is reported previously in some other metabolic disorders that result in pathologic accumulation of metabolites or toxic brain damage. Therapy with GnRH agonist triptorelin effectively arrested pubertal development.

## Highlights

• Girl with 3-MGA-I presented with central precocious puberty.
• Novel *AUH* gene mutation and almost undetectable 3-MGH-enzyme activity were detected.
• GnRH agonist triptorelin effectively arrested pubertal development.
• Precocious puberty is reported in some other metabolic disorders.

## 1 Introduction

3-methylglutaconic aciduria type 1 (3-MGA-I) (MIM ID #250950) is an ultra-rare, autosomal recessive organic aciduria [1]. The 3-MGA-I results from mutated *AUH* gene, encoding the 3-methylglutaconyl-CoA hydratase (3-MGH). 3-MGH catalyzes one of the steps of leucine degradation - the conversion of 3-methylglutaconyl-CoA to 3-hydroxy-3-methylglutaryl-CoA [2,3]. Consequently, markedly increased urinary excretion of 3-methylglutaconic acid (3-MGA), and mildly increased urinary 3-methylglutaric acid (3-MG) and 3-hydroxyisovaleric acid (3-HIVA) are characteristic for 3-MGA-I and could be measured with urinary organic acids analysis [[4], [5], [6]].

The clinical spectrum of 3-MGA-1 is heterogeneous. In childhood, it varies from asymptomatic individuals to mild neurological impairment, speech delay, quadriplegia, dystonia, choreoathetoid movements, severe encephalopathy, psychomotor retardation, basal ganglia involvement [2,[4], [5], [6]]. In adult-onset condition, slowly progressive leukoencephalopathy with progressive dementia, ataxia and spasticity is reported [5].

Early dietary treatment with leucine restriction and carnitine supplementation may be effective in improving neurological state in pediatric patients with 3-MGA-1 [1,5]. Thus, 3-MGA-1 is included in some of the newborn screening (NBS) panels (secondary panel in the USA: https://www.hrsa.gov/advisory-committees/heritable-disorders/rusp/index.html) or is detected as a secondary NBS disorder [7].

Besides 3-MGA-I, four other inherited disorders are associated with excessive excretion 3-MGA in the urine but are caused by mutations in other genes: Barth syndrome (3-MGA-II, MIM# 302060) caused by a deficiency of tafazzin, Costeff optic atrophy syndrome (3-MGA-III, MIM# 258501) caused by a deficiency of unknown protein and 3-Methylglutaconic aciduria type IV and V are still not well delineated [8,9]. Patients with 3-MGA-I excrete higher level of 3-methylglutaconic acid and two metabolites 3-MG and 3-HIVA in urine as compared to patients with other MGA types [1]. All these disorders could also be detected through the NBS.

We aimed to present a girl with 3-MGA-I due to novel *AUH* gene mutation (homozygous variant c.330 + 5G > A), who initially presented with precocious puberty.

## 2 Case presentation

The 5.5-year-old girl was referred to the University Children's Hospital Ljubljana (UCHL) due to signs of precocious puberty, progressive breast development since the age of 4 years. She was the first child of presumably non-consanguineous Caucasian parents, born at the term after an uneventful pregnancy. She had one younger sibling who developed type 1 diabetes, but no other cases of early puberty were reported in the family. Maternal menarche was reported at the age of 13 years, while the father reported shaving at the age of 19 years. She had no significant perinatal history. Her early development was normal; she started walking at 11 months and there was no speech delay.

Central precocious puberty was confirmed at her first visit by elevated luteinizing hormone (LH) values (basal LH 0.2 IU/L, peak LH 26.2 IU/L after stimulation with GnRH 100 μg/m^2^ body surface) significantly advanced bone age (+3.5 SD) and increased growth velocity (she gained 6 cm of height in the 6 months before referral). Afterwards she had been regularly every 3 months receiving therapy with GnRH agonist triptorelin, which effectively arrested pubertal development. At the age of 6 years she had performed brain MRI to exclude central causes of precocious puberty. A MRI scan of the brain revealed T2 and FLAIR hyper-intensive lesions in centrum semiovale, bilateral subcortical frontal white matter and in the deep frontoparietal white matter. No visible changes were observed in the hypothalamic regions or pituitary.

Since the lesions on MRI were suspected to be ischemic, she was referred to the Department of Pediatric Neurology. Neurologic examination at the age of 6 years and 8 months revealed central hypotonia, intention tremor and dysdiadochokinesia. There was no other significant finding in systemic physical examination. Due to suspicion of ischemic lesions on MRI a panel of tests for hypercoagulability were performed and were normal. An electrocardiogram, echocardiogram and trans-cranial Doppler and the examinations were all normal.

She had also performed selective metabolic screening where urine organic acid analysis by gas chromatography–mass spectrometry (GC/MS) revealed high levels of 3-MGA and 3-HIVA. Acylcarnitine analysis from dried blood spot was done with tandem mass spectrometry (LC-MS/MS) and showed elevated levels of 3-hydroxyisovaleryl-carnitine (C5-OH). Other metabolic panels showed normal results.

Later the 3-MGH-enzyme activity in lymphocytes was assessed (it was commercially performed in the Amsterdam University Medical Center), which showed extremely low levels (0.02 nmol/min/mg protein; reference range 1.4–4.6 nmol/min/mg protein). To confirm the diagnosis, *AUH* mutation analysis was performed; a novel homozygous variant c.330 + 5G > A was revealed in the *AUH* gene.

During the follow up, the patient had severely elevated 3-MGA in all measurements and elevated 3-HIVA. The patient had also moderately elevated C5-OH (1.5–3.2 times above upper limit; reference range 0–0.43 μmol/L) with normal total and free carnitine levels. At the time of patient birth, NBS was not performed in Slovenia. Screening card was analyzed retrograde and results did not reveal elevation of C5OH. It can be explained by the fact that the card was stored at room temperature for more than 5 years.

After the diagnosis of 3-MGA-I was confirmed, restricted leucine diet (60 mg/kg/day) and l-carnitine supplementation (85 mg/kg/day) were introduced at the age of 8 years.

Parents also reported that she was identified with attention-deficit/hyperactivity disorder at the age of 5 years and with learning disability at age of 8 years at school. Psycho-educational assessment (Beery-Buktenica Developmental Test of Visual-Motor Integration; The Tower of London test (ToL) performed by a psychologist at age 8.5 years revealed low cognitive functioning, low visual motor integration and low processing speed. In the Brown Attention-Deficit Disorder Scale for Children, she showed moderate to significant problems. Due to the learning disability, she was transferred to special education school.

**Fig. 1:**
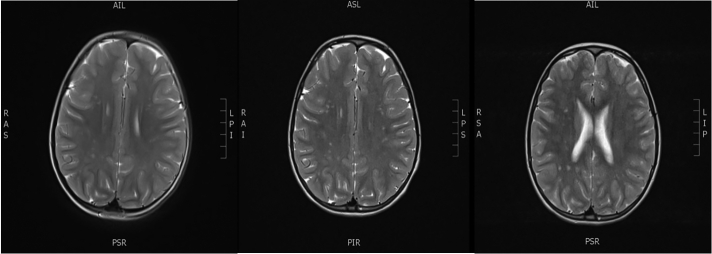
Cerebral MRI (T2-weighted) of the patient at the age of 6 years (A) shows mild abnormalities in centrum semiovale, bilateral subcortical frontal white matter and in the deep frontoparietal white matter. At the age of 9 years (B) progression of nonspecific deep and subcortical white matter signal changes are seen. MRI at the age of 10 years (C) shows no significant changes in previously described hyper-intensive lesions.

Repeat of brain MRI at age of 9 years showed progression of hyper-intensive lesions in bilateral subcortical frontal white matter and in the deep frontoparietal white matter. Aspirin Protect (100 mg/day) was introduced. Further MRI of the brain one year later showed no significant changes in previously described hyper-intensive lesions (Fig. 1).

On the last neurological examination at the age of 11 years (32 months after the leucine restricted diet) the patient showed improvement of attention. There was no deterioration in her physical and neurological examination findings compared to the baseline.

## 3 Discussion

We presented a girl with 3-MGA-I due to novel *AUH* gene splicing mutation and confirmed by almost undetectable 3-MGH-enzyme activity, who initially presented with central precocious puberty at an early age of 4.5 years.

Previously, only around 40 cases are reported. The age of onset of 3-MGA-I varied from first year of life to 52 years of age. The most common clinical feature was global developmental delay or intellectual disability in children and late onset, slowly progressive leukoencephalopathy in adults [2,[4], [5], [6],[10], [11], [12], [13], [14], [15]]. Altogether, 10 different mutations (5 missense, 3 splicing, 1 single nucleotide deletion and 1 single nucleotide duplication) have been reported in the *AUH* gene [3,5].

This is to our knowledge the first patient with 3-MGA-I and precocious puberty; furthermore, precocious puberty was a presenting sign. The co-occurrence of precocious puberty and 3-MGA-I could be coincidental; nevertheless, early onset and sporadic occurrence of precocious puberty in our patient are suggestive of organic cause. According to a recent meta-analysis, intracranial lesions are identified in 25% of girls presenting with central precocious puberty before 6 years [16]. Most common underlying pathologies are hypothalamic hamartomas; nevertheless, a wide variety of inherited or acquired brain lesions may result in precocious pubertal development [17,18]. There are anecdotic reports of patients with precocious puberty in association with various inborn errors of metabolism (IEM) inducing accumulation of toxic substances in the central nervous system. Recently, a case of a girl with metachromatic leukodystrophy with puberty starting at 4 years, similar to our case, was reported [19]. CPP was also reported in a 4 year old girl with Tay-Sachs disease, in whom GM2 gangliosides storage was described in the hypothalamus post-mortem [20], in a few patients with mucopolysaccharidosis type IIIA [21,22] and in a patient with mucopolysaccharidosis type IH [23]. A common denominator of all reported IEM is damage to the brain white matter, which was affected also in our patient. Despite various neurological symptoms, the pathophysiological mechanism of the neurotoxicity in 3-MGA-I has so far not been clearly understood. Oxidative stress is an important etiological factor in the pathogenesis of several neurodegenerative disorders as a result of an imbalance between neutralization and excessive production of reactive oxygen species [24]. Incubation of the cerebral cortex of young rats with high concentrations of 3-MGA, 3-HIVA and 3-MG in vitro induced oxidative stress. Lipid and protein oxidative damage and decreased antioxidant defense mechanisms were shown likely caused by the oxidative stress [25]. No macroscopic changes in the hypothalamus or pituitary were observed by any of brain MRI in our patient. However, it is possible that sufficient microscopic injury of the hypothalamus to weaken the suppression of the hypothalamo-pituitary-gonadal axis were induced by toxic metabolites or oxidative stress. Obviously, the extent or location of the damage did not prevent GnRH secretion, at least not in her childhood. Nevertheless, the fact that the therapy with GnRH analogue was efficient in our patient indicates the injury was upstream of the gonadotrophs.

In the pediatric group, three patients with 3-MGA-1 and attention deficit- hyperactivity disorder have been previously reported. Patient 1 was a 10-year old male patient, who presented with febrile seizures at the age of one and was diagnosed with 3-MGA-1 at the age of four and had attention deficit-hyperactivity disorder with normal cognitive functions (5, 11). Patient 2 was a 14-year old female patient who also had attention deficit-hyperactivity disorder with learning disability (6). In both patients cranial MRI showed mild abnormalities in deep frontal white matter (5, 11) and bilateral white matter changes in the subcortical frontal and parietal white matter (6). Patient 3 had in addition to shortened attention span also a retarded speech development. Unfortunately, a cranial MRI of this patient was not performed (2,3). Our patient also has mild learning disability and attention deficit-hyperactivity disorder. Her cranial MRI showed bilateral white matter changes in subcortical frontal and deep frontoparietal white matter and in centrum semiovale. These mild abnormalities in deep supratentorial white matter could represent the earliest stages of the slowly progressive leukoencephalopathy clinically presented only in adulthood. We believe the prospective follow-up of the patients with 3-MGA-1 might thus help us to learn more about the natural course of this disease.

## 4 Conclusions

To conclude, this is a first report of central precocious puberty in a girl with might be connected to the 3-MGA-I with demonstrated, as is reported previously in some other IEM, possibly due to damage in the white brain matter and additional clinical signs of central nervous system involvement. Therapy with GnRH agonist triptorelin effectively arrested pubertal development. Recognition of such specific clinical signs could participate to understanding of the pathogenesis of this ultra-rare IEM. On the other hand, revealing the mechanisms of the hypothalamic involvement in IEM patients that underwent precocious puberty could potentially participate to the understanding of the regulation of puberty.
